# Impact of inflammatory bowel disease on perioperative complications in patients undergoing surgery for metastatic spinal tumors

**DOI:** 10.3389/fmed.2026.1895624

**Published:** 2026-08-28

**Authors:** Le Fan, Kenan Sun, Wanshan Shang, Zifeng Wang, Jiayi Guo, Lanbo Yang

**Affiliations:** 1 School of Osteopathy, Henan University of Chinese Medicine, Zhengzhou, Henan, China; 2 Sports Medicine Center, Luoyang Orthopedic Hospital of Henan Province, Luoyang, Henan, China; 3 Department of Orthopedics, The Second Affiliated Hospital of Anhui Medical University, Hefei, China; 4 Institute of Orthopedics, Research Center for Translational Medicine, The Second Affiliated Hospital of Anhui Medical University, Hefei, China; 5 Division of Orthopaedic Surgery, Department of Orthopaedics, Nanfang Hospital, Southern Medical University, Guangzhou, Guangdong, China

**Keywords:** complications, database, inflammatory bowel disease, National Inpatient Sample, spinal column metastases

## Abstract

**Background:**

The global prevalence of inflammatory bowel disease (IBD) continues to rise. However, researchers have not yet investigated how IBD influences perioperative complication rates in patients undergoing surgical intervention for metastatic spinal tumors. This study used the United States National Inpatient Sample (NIS) to evaluate the association between IBD and perioperative complications in patients undergoing surgery for metastatic spinal tumors.

**Methods:**

Patients undergoing surgical treatment for spine metastases from 2016 to 2022 were identified through ICD-10-CM diagnostic coding, and their data were analyzed. The study population excluded individuals younger than 18 years. Participants were stratified according to whether they had IBD or not, and comparisons were made across demographic characteristics, hospital-related factors, intraoperative parameters, comorbid conditions, and perioperative complications. The statistical methodology encompassed Pearson χ^2^ analysis, Wilcoxon rank-sum testing, and logistic regression modeling. The perioperative complications were selected based on their clinical relevance, availability within the NIS database, and consistency with previous nationwide database studies evaluating postoperative outcomes following spine surgery.

**Results:**

Among 48,465 patients with metastatic spinal tumors 535 (1.1%) had concurrent IBD. Patients with IBD exhibited significantly higher odds of developing various perioperative complications. These included wound disruption (aOR = 5.41; *P* < 0.001), post-procedural hematoma (aOR = 4.57; *P* = 0.002), post-procedural infection (aOR = 3.83; *P* < 0.001), gastrointestinal system complications (aOR = 3.36; *P* < 0.001), and cerebrospinal fluid leakage (aOR = 1.93; *P* = 0.014).

**Conclusions:**

Patients with IBD undergoing surgery for metastatic spinal tumors were at significantly increased risk of wound-related complications, post-procedural hematoma, postoperative infection, gastrointestinal complications, and cerebrospinal fluid leakage. These findings support careful preoperative risk stratification and individualized perioperative management in this high-risk population.

## Introduction

Patients with inflammatory bowel disease (IBD) frequently present with systemic manifestations, including malnutrition, anemia, osteoporosis, and immune dysregulation, and many receive corticosteroids, immunomodulators, or biologic therapies that may adversely affect wound healing and increase susceptibility to infection (1–3). These considerations may be particularly important in patients undergoing surgery for metastatic spinal tumors, as these individuals often have advanced malignancy, impaired physiological reserve, and require complex surgical procedures with an inherently high risk of perioperative complications (4, 5).

As a systemic inflammatory disorder, IBD manifests effects that extend well beyond the gastrointestinal tract. Both intra- digestive and extra-digestive inflammatory responses can lead to functional impairments across multiple organ systems, notably affecting the immune, circulatory, nervous, and endocrine components (6–8). Yi Zhang and colleagues studied the impact of IBD on lumbar surgery and found that the surgery may increase the complexity of surgical intervention and related risks (9). One possible explanation for this is that IBD causes poor nutritional status, a well-known predictor of adverse outcomes after spine surgery (10). Tanenbaum et al. investigated the impact of IBD on lumbar spine surgery and found that IBD may lead to prolonged hospital stay and increased treatment costs, and adverse effects may also occur during surgery for a metastatic bony spinal tumor given the systemic effects of IBD (11).

However, no studies investigated the impact of IBD on perioperative complications in patients undergoing surgery for metastatic spinal tumors, nor did a large-scale database analysis be conducted. The aim of this study was to investigate the association between IBD and perioperative complications in patients undergoing surgery for metastatic spinal tumors using a large national database.

## Methods

### Data source

Data for this retrospective study were obtained from the National Inpatient Sample (NIS). This extensive database, which captures inpatient healthcare information, is maintained by the Healthcare Cost and Utilization Project under the auspices of the Agency for Healthcare Research and Quality. The NIS provides a highly representative dataset by encompassing approximately 20% of all United States hospital admissions, with data drawn from more than 1,000 participating hospitals. As the nation's largest all-payer inpatient database, it contains data on over 7 million hospital stays annually, which translates to approximately 35 million weighted hospitalizations each year (12). We collected data on all patients who received surgical intervention for metastatic spinal bone tumors during the period spanning 2016 to 2022. This was accomplished by querying specific surgical and diagnostic codes from the International Classification of Diseases, Tenth Edition (ICD-10), specifically codes C79.51 and C79.52. The compiled dataset encompassed demographic characteristics, patient comorbidities, intraoperative variables, and postoperative complications.

### Study population

**Figure 1:**
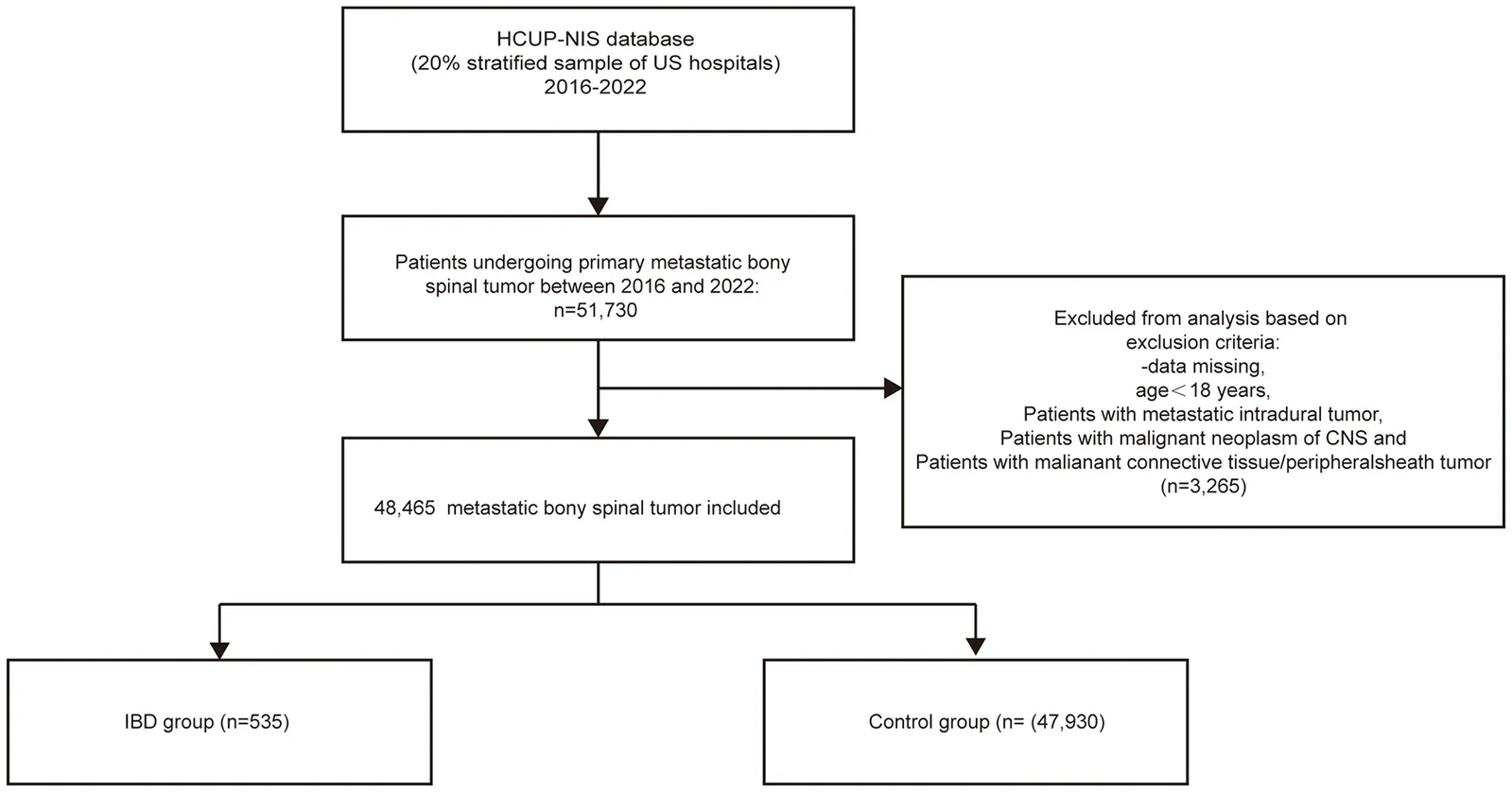
The algorithm used to identify cohorts. CNS, central nervous system.

After removing incomplete records, patients younger than 18 years, and those with diagnostic codes for central nervous system malignancies, connective tissue/peripheral sheath malignant tumors, and/or metastatic intradural neoplasms, 48,465 cases remained for final analysis (Figure 1). Patients were stratified into two cohorts according to whether they had concurrent IBD, with the condition identified via ICD-10 codes K50, K51, and K52. Baseline variables included demographic characteristics (age, sex, race, median household income quartile, and primary payer), hospital characteristics (bed size, teaching status, and geographic region), and 24 preoperative comorbidities identified using the Elixhauser Comorbidity Software Refined. Because IBD was the exposure variable of interest, it was not included among the adjustment comorbidities.

### Outcome measures

The primary outcomes were perioperative complications occurring during the index hospitalization, including cerebrospinal fluid leakage, wound disruption, post-procedural infection, post-procedural hematoma, gastrointestinal complications, pneumonia, urinary tract infection, acute kidney injury, sepsis, pulmonary embolism, deep vein thrombosis, and myocardial infarction. These complications were selected because they represent clinically important postoperative adverse events that can be reliably identified using ICD-10-CM diagnosis codes in the National Inpatient Sample and have been commonly evaluated in previous nationwide studies of spine surgery (13).

Secondary outcomes included length of hospital stay, total hospital charges, discharge disposition, and in-hospital mortality. Routine discharge was defined as discharge to home (including home health care when applicable), whereas non-routine discharge included discharge to skilled nursing facilities, rehabilitation facilities, long-term care facilities, other healthcare institutions, hospice, or transfer to another acute care hospital. Intraoperative variables were also collected as adjustment covariates. All variables listed in Table 1 were simultaneously entered into the multivariable logistic regression models, including demographic characteristics (age, sex, race, median household income quartile, and primary payer), hospital characteristics (hospital bed size, teaching status, geographic region, and hospital location), the 24 preoperative comorbidities identified using the Elixhauser Comorbidity Software Refined, and intraoperative procedures.

**Table 1:** Variables used in binary logistic regression analysis.

Variables categories	Specific variables
Patient demographics	Age, sex (male and female), race (White, Black, Hispanic, Other)
Hospital characteristics	Bed size of hospital (small, medium, large), teaching status of hospital (non-teaching, teaching), location of hospital (rural, urban), type of insurance (Medicare, Medicaid, private insurance, other), location of the hospital (northeast, Midwest or north central, south, west), Income quartile (0–25th, 26–50th, 51–75th, 76–100th)
Comorbidities	Nicotine dependence, Opioid dependence, Deficiency anemia, Rheumatoid arthritis/collagen vascular diseases, Chronic blood loss anemia, Congestive heart failure, Chronic pulmonary disease, Coagulopathy, Diabetes, uncomplicated, Drug abuse, Hypertension, Hypothyroidism, Liver disease, Lymphoma, Fluid and electrolyte disorders, Other neurological disorders, Obesity, Peripheral vascular disorders, Pulmonary circulation disorders, Renal failure, Valvular disease, Weight loss, Peptic ulcer disease, Alcohol abuse
Complications	Cerebrospinal fluid leak or dural tear, Pressure ulcer, Post-procedural fever, Acute post-hemorrhagic anemia, Displacement of internal fixation device of vertebrae, Post-procedural shock, Wound disruption, Post-procedural infection, Mechanical device complication, Post-procedural hematoma, Digestive system complications, Urinary tract infection
Intraoperative variables	Electrophysiologic monitoring, blood transfusion, Platelet transfusion, Excision of spinal meninges/cord, Release spinal meninges/cord, Excision spine vertebra, Cervical fusion, Cervicothoracic fusion, Thoracic fusion, Thoracolumbar fusion, Lumbar fusion, Lumbosacral fusion

AIDS, Acquired immunodeficiency syndrome.

### Data analysis

Statistical analyses were performed using R version 3.5.3 (R Foundation for Statistical Computing, Vienna, Austria) and IBM SPSS Statistics version 25.0 (IBM Corp., Armonk, NY, USA). A two-sided α = 0.05 was defined as the threshold for statistical significance (*P* < 0.05).

Missing data were handled using a combination of complete-case analysis and categorical retention, depending on the variable. Patients with missing values for variables essential for cohort definition, exposure classification, outcome ascertainment, or multivariable adjustment were excluded from the final analytic cohort. For variables in the NIS database with predefined “missing” categories (e.g., race and median household income quartile), missing values were retained as separate categories in the analyses. Multiple imputation was not performed because missingness was limited and the NIS database requires standardized reporting for most core demographic and hospitalization variables.

Continuous variables were presented as mean ± standard deviation (SD) or median with interquartile range (IQR), as appropriate, whereas categorical variables were presented as frequencies and percentages. Comparisons between groups were performed using the independent Student's *t*-test or Mann–Whitney U test for continuous variables and Pearson's chi-square test for categorical variables. Descriptive analyses were conducted to summarize baseline characteristics of the study population. Multivariable logistic regression analyses were performed to evaluate the association between IBD and each perioperative complication among patients who underwent surgical intervention for spinal metastatic bone tumors. Multivariable logistic regression analyses were performed to evaluate the association between IBD and each study outcome. Separate regression models were constructed for each primary and secondary outcome. All covariates listed in Table 1 were simultaneously entered into the multivariable logistic regression models.

## Results

### Patient demographics and hospital characteristics

Among the 48,465 patients analyzed, IBD was identified in 535 cases (1.1%). Notable demographic disparities emerged when comparing patients with IBD to those without this comorbidity. The IBD population demonstrated several distinct demographic and enrollment characteristics relative to patients without IBD (Table 2). Specifically, white patients comprised a significantly higher percentage of the IBD cohort (75.7% vs. 68.5%; *P* < 0.001). Medicare enrollment was also more prevalent among IBD patients (51.4% vs. 47.8%; *P* < 0.001). Furthermore, individuals with IBD received care at Midwest facilities with greater frequency (29.0% vs. 20.9%; *P* < 0.001).

**Table 2:** Patient characteristics and outcomes after Surgery for metastatic spinal tumors (2016–2022).

Characteristics	No-IBD	IBD	*P*
Total (*n* = count)	47,930	535	
Total incidence (%)	1.1		
Age (median, years)	64.0 (57.0, 72.0)	63.0 (55.0, 72.0)	0.512
Gender (%)			
Male	59.9	58.9	0.635
Female	40.1	41.1	
Race (%)			
White	68.5	75.7	< 0.001
Black	14.0	15.9	
Hispanic	9.3	4.7	
Other	8.1	3.7	
Income quartile (%)			
0–25th	24.1	21.5	0.209
26–50th	24.3	22.4	
51–75th	25.2	28.0	
76–100th	26.4	28.0	
Type of insure (%)			
Medicare	47.8	51.4	< 0.001
Medicaid	12.7	6.5	
Private insurance	33.9	37.4	
Other	5.6	4.7	
Bed size of hospital (%)			
Small	9.0	9.3	0.537
Medium	21.6	19.6	
Large	69.4	71.0	
Region of hospital (%)			
Northeast	20.9	21.5	< 0.001
Midwest or North Central	20.9	29.0	
South	36.3	26.2	
West	21.9	23.4	
Hospital type (%)			
Rural	1.2	0.9	0.127
Urban non-teaching	9.0	6.5	
Urban teaching	89.9	92.5	
TOTCHG (median, $)	193, 815.0 (121, 058.0–310, 240.0)	200, 400.0 (120, 053.0–339, 857.0)	0.022
LOS (median, d)	9.0 (5.0–14.0)	10.0 (7.0–18.0)	< 0.001

### Admission and patient comorbidities

Compared with patients without IBD, patients with IBD demonstrated a higher prevalence of several preoperative comorbidities. Specifically, patients with IBD had higher rates of rheumatoid arthritis or collagen vascular disease (2.8% vs. 1.2%, *P* = 0.001), chronic pulmonary disease (20.6% vs. 17.3%, *P* = 0.046), coagulopathy (14.0% vs. 11.3%, *P* = 0.048), hypothyroidism (15.9% vs. 12.3%, *P* = 0.012), lymphoma (10.3% vs. 3.9%, *P* < 0.001), fluid and electrolyte disorders (42.1% vs. 29.5%, *P* < 0.001), other neurological disorders (12.1% vs. 8.7%, *P* = 0.006), renal failure (14.0% vs. 9.4%, *P* < 0.001), weight loss (31.8% vs. 17.6%, *P* < 0.001), and peptic ulcer disease (1.9% vs. 0.5%, *P* < 0.001) (Table 3).

**Table 3:** Relationship between inflammatory bowel disease and preoperative comorbidities.

Comorbidities	Univariate analysis		
	No-IBD	IBD	*P*
Nicotine dependence	7,260 (15, 1%)	95 (17.8%)	0.094
Opioid dependence	750 (1.6%)	10 (1, 9%)	0.573
Deficiency anemia	2,135 (4.5%)	25 (4.7%)	0.808
Rheumatoid arthritis/collagen vascular diseases	585 (1.2%)	15 (2.8%)	0.001
Chronic blood loss anemia	505 (1.1%)	5 (0.9%)	0.788
Congestive heart failure	2,805 (5.9%)	25 (4.7%)	0.247
Chronic pulmonary disease	8,280 (17.3%)	110 (20.6%)	0.046
Coagulopathy	5,415 (11.3%)	75 (14.0%)	0.048
Diabetes, uncomplicated	4,400 (9.2%)	30 (5.6%)	0.004
Drug abuse	1,295 (2.7%)	10 (1.9%)	0.237
Hypertension	27,600 (57.6%)	330 (61.7%)	0.056
Hypothyroidism	5,895 (12.3%)	85 (15.9%)	0.012
Liver disease	2,755 (5.7%)	35 (6.5%)	0.433
Lymphoma	1,860 (3.9%)	55 (10.3%)	< 0.001
Fluid and electrolyte disorders	14,135 (29.5%)	225 (42.1%)	< 0.001
Other neurological disorders	65 (12.1%)	4,190 (8.7%)	0.006
Obesity	6,905 (14.4%)	55 (10.3%)	0.007
Peripheral vascular disorders	2,200 (4.6%)	15 (2.8%)	0.049
Pulmonary circulation disorders	650 (1.4%)	5 (0.9%)	0.401
Renal failure	4,505 (9.4%)	75 (14.0%)	< 0.001
Valvular disease	1,485 (3.1%)	10 (1.9%)	0.102
Weight loss	8,450 (17.6%)	170 (31.8%)	< 0.001
Peptic ulcer disease	230 (0.5%)	10 (1.9%)	< 0.001
Alcohol abuse	1,190 (2.5%)	5 (0.9%)	0.022

### Intraoperative procedures and variables

The proportion of each surgical procedure type was similar between the cohorts, with the most common procedures in both cohorts being blood transfusion (No- IBD, 14.6% vs. IBD, 15.0%; *P* = 0.862), followed by excision of lumbosacral fusion (No- IBD, 4.9% vs. IBD, 5.6%; *P* = 0.418) and release platelet transfusion (No- IBD, 2.9% vs. IBD, 3.7%; *P* = 0.238) (Table 4).

**Table 4:** Intraoperative procedures and variables.

Variables (%)	Univariate analysis		
	No-IBD	IBD	P
Blood transfusion	7,005 (14.6%)	80 (15.0%)	0.826
Cervical fusion	8,195 (17.1%)	80 (15.0%)	0.190
Cervicothoracic fusion	4,735 (9.9%)	45 (8.4%)	0.257
Excision of spinal meninges/cord	12,290 (25.6%)	140 (26.2%)	0.781
Excision spine vertebra	13,625 (28.4%)	135 (25.2%)	0.103
Electrophysiologic monitoring	13,495 (28.2%)	175 (32.7%)	0.020
Lumbar fusion	9,615 (20.1%)	100 (18.7%)	0.432
Lumbosacral fusion	2,325 (4.9%)	30 (5.6%)	0.418
Platelet transfusion	1,380 (2.9%)	20 (3.7%)	0.238
Release spinal meninges/cord	18,595 (38.8%)	175 (32.7%)	0.004
Thoracic fusion	21,425 (44.7%)	265 (49.5%)	0.025
Thoracolumbar fusion	6,185 (12.9%)	160 (11.2%)	0.246

### Perioperative complications associated with IBD

**Figure 2:**
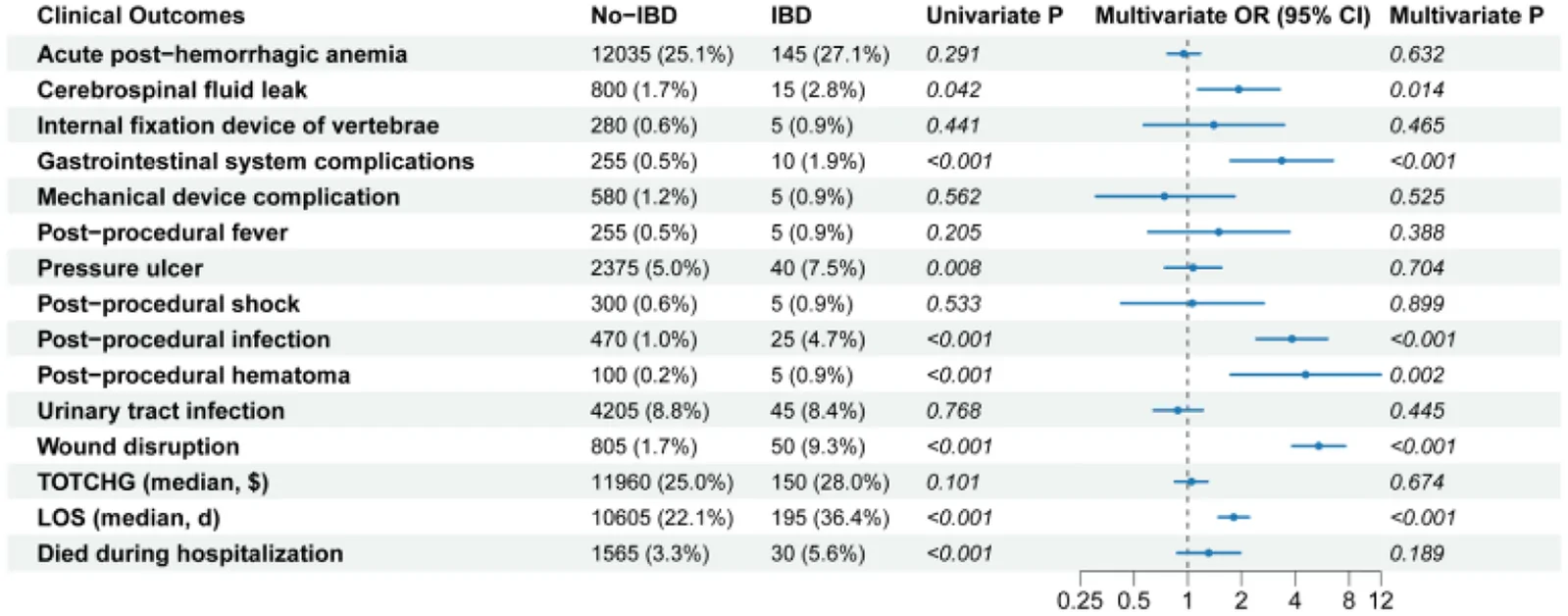
Relationship between inflammatory bowel disease and postoperative complications.

Among patients with IBD undergoing surgical intervention for metastatic spinal tumors, several postoperative complications occurred at higher rates compared with patients without IBD. Specifically, patients with IBD demonstrated increased incidences of cerebrospinal fluid leakage (2.8% vs. 1.7%, *P* = 0.042), gastrointestinal system complications (1.9% vs. 0.5%, *P* < 0.001), pressure ulcers (7.5% vs. 5.0%, *P* = 0.008), post-procedural infection (4.7% vs. 1.0%, *P* < 0.001), post-procedural hematoma (0.9% vs. 0.2%, *P* < 0.001), and wound disruption (9.3% vs. 1.7%, *P* < 0.001) compared with non-IBD patients (Table 5). Although pressure ulcers were more frequent among patients with IBD in the univariate analysis (7.5% vs. 5.0%, *P* = 0.008), this association was attenuated and no longer significant after multivariate adjustment (adjusted OR = 1.07, 95% CI: 0.747–1.541, *P* = 0.704). After adjustment for all covariates listed in Table 1, IBD remained independently associated with wound disruption (aOR = 5.41, 95% CI: 3.84–7.62), postoperative infection (aOR = 3.83, 95% CI: 2.43–6.04), postoperative hematoma (aOR = 4.57, 95% CI: 1.74–12.00), cerebrospinal fluid leakage (aOR = 1.93, 95% CI: 1.14–3.27), gastrointestinal complications (aOR = 3.36, 95% CI: 1.74–6.49), and prolonged hospitalization (aOR = 1.81, 95% CI: 1.49–2.20) (Figure 2).

**Table 5:** Relationship between inflammatory bowel disease and postoperative complications.

Complications	Univariate analysis			Multivariate logistic regression		
	No-IBD	IBD	*P*	aOR	95% CI	*P*
Acute post-hemorrhagic anemia	12,035 (25.1%)	145 (27.1%)	0.291	0.95	0.771–1.171	0.632
Cerebrospinal fluid leak	800 (1.7%)	15 (2.8%)	0.042	1.93	1.139–3.269	0.014
Displacement of internal fixation device of vertebrae	280 (0.6%)	5 (0.9%)	0.441	1.40	0.567–3.460	0.465
Gastrointestinal system complications	255 (0.5%)	10 (1.9%)	< 0.001	3.36	1.736–6.492	< 0.001
Mechanical device complication	580 (1.2%)	5 (0.9%)	0.562	0.74	0.307–1.828	0.525
Post-procedural fever	255 (0.5%)	5 (0.9%)	0.205	1.49	0.601–3.712	0.388
Pressure ulcer	2,375 (5.0%)	40 (7.5%)	0.008	1.07	0.747–1.541	0.704
Post-procedural shock	300 (0.6%)	5 (0.9%)	0.533	1.06	0.424–2.659	0.899
Post-procedural infection	470 (1.0%)	25 (4.7%)	< 0.001	3.83	2.428–6.041	< 0.001
Post-procedural hematoma	100 (0.2%)	5 (0.9%)	< 0.001	4.57	1.741–12.000	0.002
Urinary tract infection	4,205 (8.8%)	45 (8.4%)	0.768	0.88	0.645–1.212	0.445
Wound disruption	805 (1.7%)	50 (9.3%)	< 0.001	5.41	3.844–7.624	< 0.001
TOTCHG (median, $)	11,960 (25.0%)	150 (28%)	0.101	1.05	0.848–1.289	0.674
LOS (median, d)	10,605 (22.1%)	195 (36.4%)	< 0.001	1.81	1.490–2.201	< 0.001
Died during hospitalization	1,565 (3.3%)	30 (5.6%)	< 0.001	1.31	0.875–1.968	0.189
Discharge disposition (%)						
Routine	23,275 (48.6%)	240 (44.9)	0.011	0.90	0.824–1.187	0.989
Non-routine	23,010 (48.0%)	265 (49.5%)				
Other	1,645 (3.4%)	30 (5.6%)				

OR, Odds ratio; CI, Confidence interval.

### Association between IBD and clinical outcomes

**Figure 3:**
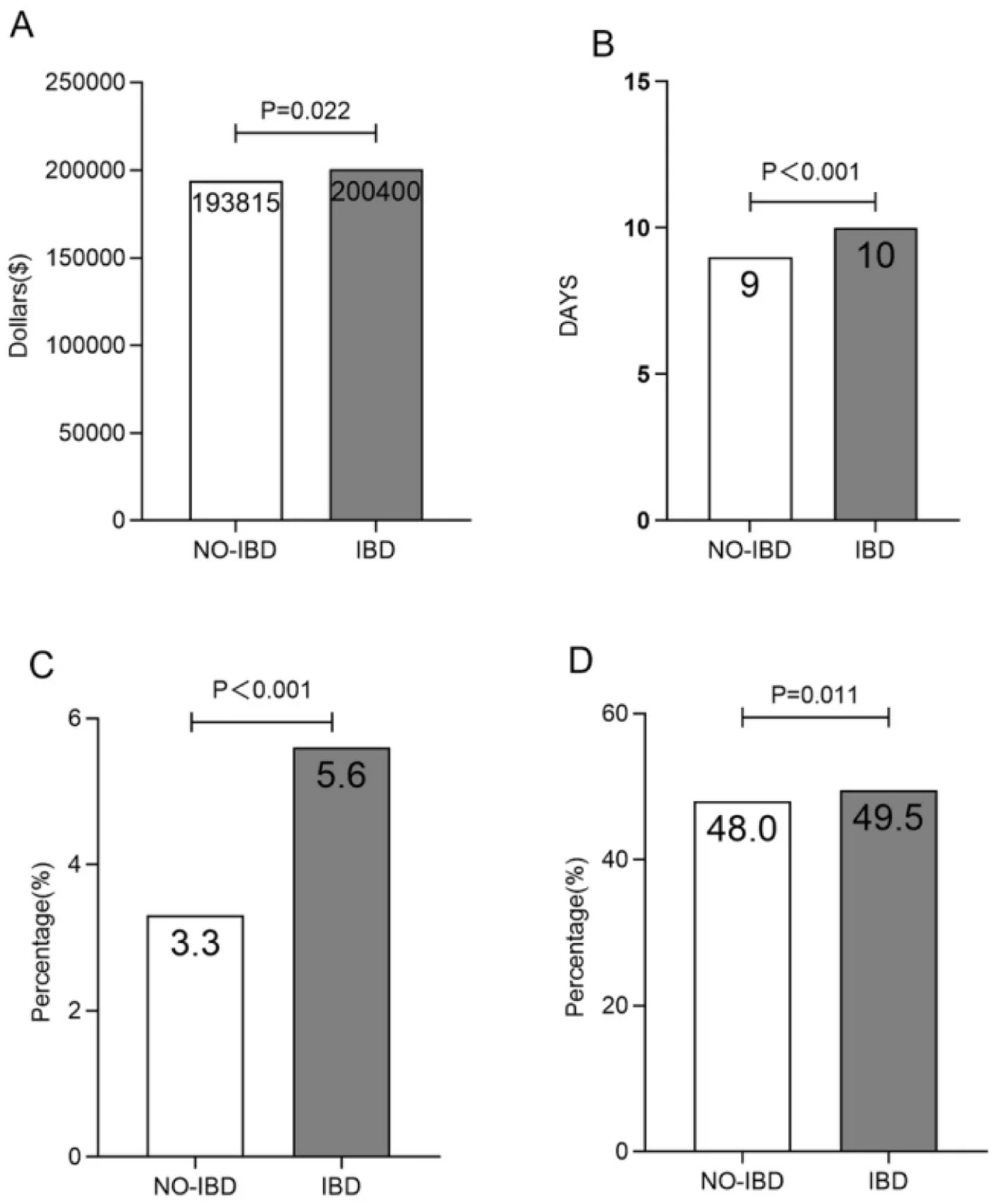
Comparison of No Inflammatory Bowel Disease (No-IBD) cohort and Inflammatory Bowel Disease (IBD) cohort. **(A)** Total cost of admission. **(B)** Length of stay. **(C)** Perioperative mortality rates. **(D)** Discharge disposition.

In addition to postoperative complications, we further evaluated the association between inflammatory bowel disease (IBD) and overall clinical outcomes following surgical intervention for metastatic spinal tumors. Compared with patients without IBD, patients with IBD experienced significantly longer hospital stays, with a higher proportion of prolonged hospitalization observed in the IBD cohort (36.4% vs. 22.1%, *P* < 0.001). This association remained statistically significant after multivariate adjustment (adjusted OR [aOR] =1.81, 95% CI: 1.490–2.201, *P* < 0.001). Although patients with IBD demonstrated higher in-hospital mortality compared with non-IBD patients (5.6% vs. 3.3%, *P* < 0.001), multivariate logistic regression analysis showed that IBD was not independently associated with increased mortality risk (aOR = 1.31, 95% CI: 0.875–1.968, *P* = 0.189). Similarly, although differences in discharge disposition were observed between the two groups in univariate analysis (*P* = 0.011), no significant association remained after adjustment (aOR = 0.90, 95% CI: 0.824–1.187, *P* = 0.989). Furthermore, no significant difference was observed in total hospital charges between patients with and without IBD (28.0% vs. 25.0%, *P* = 0.101; aOR = 1.05, 95% CI: 0.848–1.289, *P* = 0.674) (Table 6; Figure 3).

**Table 6:** Relationship between inflammatory bowel disease and clinical outcomes following surgery for metastatic spinal tumors.

Complications	Univariate analysis			Multivariate logistic regression		
	No-IBD	IBD	*P*	aOR	95% CI	*P*
High healthcare cost	11,960 (25.0%)	150 (28%)	0.101	1.05	0.848–1.289	0.674
Prolonged length of stay	10,605 (22.1%)	195 (36.4%)	< 0.001	1.81	1.490–2.201	< 0.001
Died during hospitalization	1,565 (3.3%)	30 (5.6%)	< 0.001	1.31	0.875–1.968	0.189
Discharge disposition (%)					
Routine	23,275 (48.6%)	240 (44.9)	0.011	0.90	0.824–1.187	0.989
Non-routine	23,010 (48.0%)	265 (49.5%)				
Other	1,645 (3.4%)	30 (5.6%)				

OR, Odds ratio; CI, Confidence interval.

## Discussion

In this large nationwide cohort of 48,465 patients undergoing surgery for metastatic spinal tumors, IBD was present in 1.1% of cases and was independently associated with significantly worse perioperative outcomes across multiple domains. After adjustment for a comprehensive set of patient, hospital, and surgical covariates, IBD conferred a markedly elevated risk of wound disruption (aOR = 5.41), postoperative hematoma (aOR = 4.57), postoperative infection (aOR = 3.83), gastrointestinal complications (aOR = 3.36), and cerebrospinal fluid leakage (aOR = 1.93). These complications translated into substantially longer hospital stays, higher total charges, and notably increased in-hospital mortality (5.6% vs. 3.3%). To our knowledge, this represents the first systematic large-scale analysis of IBD as a risk modifier in this specific surgical population, extending prior observations from lumbar spine surgery to the more complex setting of metastatic disease.

Regarding demographic characteristics, the higher proportion of White patients and patients from the Midwest region among those with IBD was consistent with previous epidemiological studies demonstrating racial and geographic variations in IBD prevalence in the United States (14–16). In contrast, the higher proportion of Medicare beneficiaries in the IBD cohort may reflect an older population with greater comorbidity burden and more complex healthcare needs. Previous studies among Medicare populations have shown that IBD is increasingly prevalent among older adults and that insurance status is closely associated with healthcare utilization and resource consumption (17, 18). These demographic differences may partially reflect the underlying clinical complexity of patients with IBD and provide important context for interpreting their increased perioperative burden.

Regarding comorbidity burden, our study showed that patients with IBD were more likely to have fluid and electrolyte disorders, significant weight loss, coagulopathy, chronic pulmonary disease, and renal dysfunction. These findings suggest that, before undergoing surgery for spinal metastases, many of these patients were already in a state of systemic inflammation, nutritional depletion, and reduced organ reserve. Chronic diarrhea, mucosal barrier injury, and malabsorption in IBD can directly lead to fluid and electrolyte imbalance (19). Meanwhile, persistent inflammatory activity may further aggravate poor nutritional intake, protein-energy wasting, and sarcopenia, making weight loss not only a disease manifestation but also a marker of increased perioperative vulnerability (6, 20, 21). In addition, the presence of coagulopathy in IBD likely reflects not simply a bleeding tendency, but rather a broader dysregulation of the inflammation-coagulation axis. This imbalance is especially important in the setting of spinal tumor surgery, where it may interact with the risk of postoperative hematoma formation (22). Similarly, although renal and pulmonary involvement may be clinically underappreciated, both can further compromise preoperative physiological reserve and increase the complexity of perioperative management (23, 24). Taken together, these comorbidities are not independent phenomena; instead, they reflect the overall frailty of patients with IBD and provide a plausible pathophysiological basis for the higher rates of wound dehiscence, infection, and hematoma observed in this study. Kodia et al. (25) also reported that malnutrition in patients with IBD was closely associated with infection, bleeding, and prolonged hospital stay, which is consistent with the elevated complication burden observed here (26).

Our results further showed that IBD was associated with significantly higher risks of surgical wound dehiscence, postoperative infection, postoperative hematoma, gastrointestinal complications, and cerebrospinal fluid leak. Of note, the markedly increased risk of wound disruption observed in our study (aOR = 5.41) appeared greater than that reported in previous studies of IBD patients undergoing elective lumbar surgery (27). This difference may reflect the combined influence of IBD-related physiological vulnerability and the greater complexity of metastatic spinal tumor surgery, including extensive tumor resection and spinal stabilization procedures. IBD is often accompanied by malnutrition, micronutrient deficiencies, and hypoalbuminemia, all of which impair collagen synthesis and tissue repair, potentially leading to postoperative wound non-healing (19, 20, 28). In addition, perioperative exposure to immunomodulatory therapies, including corticosteroids, may further increase the risk of infection. Prevention of postoperative infection in spine surgery also emphasizes that preoperative nutritional optimization is a key strategy for reducing infectious complications (6, 27). The increased risk of postoperative hematoma may be partly related to inflammation-associated coagulation dysfunction and the prothrombotic state seen in IBD. Previous studies have demonstrated clear abnormalities in hemostatic pathways and an increased risk of thrombosis in patients with IBD (22). Hematoma formation may also be influenced by prior anticoagulant use, the anatomical complexity of spinal surgery, intraoperative blood loss, and local hemostatic strategies. Gastrointestinal complications may be more common in patients with IBD because of their underlying disease susceptibility, and this risk may be further amplified by perioperative stress, anesthesia, fasting, postoperative opioid analgesia, and impaired bowel motility, all of which can delay recovery of gastrointestinal function (29, 30). The mechanism underlying the elevated risk of cerebrospinal fluid leakage is less well established. Possible contributing factors include connective tissue fragility related to chronic nutritional deficiency, increased surgical complexity associated with higher comorbidity burden, and greater rates of adhesion or anatomical distortion from tumor involvement. Given the relatively modest effect size (aOR = 1.93) compared with other complications, this association warrants cautious interpretation and prospective validation (31).

This study also found that patients with IBD had longer hospital stays, higher costs, and higher in-hospital mortality, suggesting that their poor outcomes were driven mainly by greater baseline frailty, a heavier burden of postoperative complications, and reduced physiological reserve (32). Prior studies have shown that when patients with IBD are also frail, both LOS and hospitalization costs increase substantially (33). In patients undergoing surgery for spinal metastases, frailty has similarly been associated with longer hospitalization, more non-routine discharge, and higher total costs, indicating that poor baseline health can significantly amplify resource utilization in this setting (13, 33). Although IBD patients were more likely to experience non-routine discharge (49.5% vs. 48.0%; *P* = 0.011), the absolute difference was modest and should be interpreted with caution, as the statistical significance may be partly attributable to the large sample size rather than a clinically meaningful distinction (34). The near doubling of in-hospital mortality in IBD patients (5.6% vs. 3.3%) represents the most clinically consequential finding of the present study. This excess mortality likely reflects a convergence of multiple adverse factors: higher baseline comorbidity burden, greater susceptibility to serious perioperative complications (particularly infection and hematoma), and reduced physiological reserve to withstand surgical stress. Whether this mortality signal persists beyond the index hospitalization, and whether it is modifiable through preoperative optimization, remains an important question for future prospective research (35–37). Therefore, our findings suggest that IBD is not only associated with a higher risk of complications, but may also serve as an adverse prognostic marker in patients undergoing surgery for spinal metastases.

## Study limitations

This study has several limitations that should be considered when interpreting the findings. First, because this was a retrospective study based on the NIS administrative database, several clinically important variables were unavailable, including IBD severity, disease duration, disease activity, nutritional status, laboratory parameters, medication exposure, and functional status. Although multivariable adjustment was performed for a broad range of demographic characteristics, comorbidities, hospital characteristics, and surgical factors, residual confounding from unmeasured variables cannot be completely excluded. Therefore, the observed associations may partly reflect differences in disease severity and perioperative management rather than the diagnosis of IBD alone. Accordingly, our findings should be interpreted as demonstrating associations rather than direct causal relationships. Second, the NIS database captures only inpatient encounters; therefore, post-discharge complications, readmissions, and long-term outcomes could not be evaluated. In particular, mortality in this study represents only in-hospital death and does not reflect long-term survival. Third, although IBD patients represented a relatively small proportion of the overall cohort, this reflects the low prevalence of IBD among patients undergoing surgery for metastatic spinal tumors. The limited number of IBD cases may reduce statistical power for detecting modest associations; however, the large overall sample size and multivariable adjustment strengthen the reliability of the observed findings. Finally, because the NIS relies on ICD-10-CM diagnosis and procedure codes, potential misclassification or incomplete capture of certain variables cannot be completely excluded. Nevertheless, the use of standardized coding algorithms and the large nationally representative sample likely minimize the impact of such limitations on the overall conclusions.

## Clinical significance

These findings have important clinical implications for the perioperative management of IBD patients undergoing surgery for metastatic spinal tumors. Because these patients are more likely to present with nutritional depletion, coagulation abnormalities, immune dysregulation, and multiple comorbidities before surgery, preoperative risk stratification should not focus solely on the spinal lesion itself, but should also include a comprehensive assessment of nutritional status, coagulation function, and immune status. When abnormalities are identified, proactive optimization of nutrition and correction of fluid-electrolyte imbalance should be performed before surgery whenever possible. In addition, perioperative immunosuppressive therapy should be used judiciously and individualized to balance disease control with infection risk. Given the complexity of these patients, multidisciplinary management involving spine surgeons, gastroenterologists, nutrition specialists, anesthesiologists, and rehabilitation teams may help reduce wound complications, infection, hematoma, and delayed recovery, ultimately improving perioperative outcomes.

## Conclusion

In this large nationwide study, IBD was associated with worse perioperative outcomes in patients undergoing surgery for metastatic spinal tumors. Among the observed complications, wound dehiscence, postoperative infection, and postoperative hematoma were the most prominent adverse events and should be considered the key perioperative concerns in this population. In addition, IBD was associated with longer hospitalization, higher in-hospital mortality, and increased healthcare costs, indicating a substantially greater perioperative burden. These findings indicate that IBD is an important adverse prognostic factor in spinal metastasis tumor surgery, and the specific risks of wound dehiscence, infection, and bleeding-related complications should be more closely monitored in this vulnerable population.

## Data Availability

The original contributions presented in the study are included in the article/supplementary material, further inquiries can be directed to the corresponding authors.
